# Phylogenomics and phylodynamics of SARS-CoV-2 genomes retrieved from India

**DOI:** 10.2217/fvl-2020-0243

**Published:** 2020-11-30

**Authors:** Sameera Farah, Ashwin Atkulwar, Manas Ranjan Praharaj, Raja Khan, Ravikumar Gandham, Mumtaz Baig

**Affiliations:** 1 ^1^Department of Zoology, Laboratory of Molecular & Conservation Genetics, Govt. Vidarbha Institute of Science & Humanities, VMV, Road, Amravati 444604, India; 2 ^2^Department of Integrative Biology, University of Guelph, Guelph, 50 Stone Road, East, N1G 2W1, ON, Canada; 3 ^3^Department of Zoology, Amolakchand Mahavidyalaya, Godhani Road, Yavatmal 445001, India; 4 ^4^National Institutes of Animal Biotechnology, Gowlidoddy, Hyderabad, Telangana 500032, India; 5 ^5^Division of Veterinary Biotechnology, ICAR-Indian Veterinary Research Institute (IVRI), Izatnagar, Bareilly, Uttar Pradesh, India

**Keywords:** India, phylodynamics, SARS-nCoV-2

## Abstract

**Background:** This is the first phylodynamic study attempted on SARS-CoV-2 genomes from India to infer the current state of severe acute respiratory syndrome coronavirus 2 (SARS-CoV-2) evolution using phylogenetic network and growth trends. **Materials & Methods:** Out of 286 retrieved whole genomes from India, 138 haplotypes were used to build a phylogenetic network. The birth–death serial model (BDSIR) package of BEAST2 was used to calculate the reproduction number of SARS-CoV-2. Population dynamics were investigated using the stamp date method as implemented in BEAST2 and BEAST 1.10.4. **Results:** A median-joining network revealed two ancestral clusters. A high basic reproduction number of SARS-CoV-2 was found. An exponential rise in the effective population size of Indian isolates was detected. **Conclusion:** The phylogenetic network reveals dual ancestry and possibility of community transmission of SARS-CoV-2 in India.

Throughout human history, viral outbreaks have been an omnipotent threat to global human health from the spread of the Spanish flu epidemic in 1918 to the recent corona virus (CoV) pandemic in December 2019 [1–3]. CoVs are members of family *Coronaviridae* with a characteristic crown-like structure and have a positive-sense ssRNA genome of 26–32 kb, while SARS-CoV-2 has a genome of 29,844–29,891 nt [4–6]. The prevalence of CoVs has been reported previously in livestock and wild species [7–10]. Their rapidly evolving nature makes them capable of adapting and infecting a range of hosts with broad tissue tropism [11,12]. The first corona viral outbreak – porcine epidemic diarrhea – was reported in pigs in Europe and Asia in 1971 [8]. However, the zoonotic shift of CoVs in humans began with the worldwide outbreak of SARS-CoV-1 in China during 2002–2003 and subsequently with the Middle East respiratory syndrome in 2012 [13–15]. In the current ongoing epidemic, the first case of the novel CoV, caused by the new strain of CoVs, in other words, SARS-CoV-2, was reported from the Wuhan city of China on 31 December 2019 and spread worldwide by January 2020 [16,17]. The WHO declared this outbreak as a global pandemic on 11 March 2020. The novel CoV strain is characterized primarily by its potential to infect the human respiratory tract, causing severe pneumonia in the affected person. Whole-genome sequencing and phylogenetic analysis suggests that bats might be the original hosts [5]. Several studies, however, point toward the *S gene* of SARS-CoV-2 exhibiting high similarity of functional domains with isolates from the pangolin [18–20]. Advances in computational genomics and the availability of genomic data enable us to understand the spatial distribution and epidemiology of pandemics. The whole-genome sequencing and annotation of a novel SARS-CoV-2 reference genome opens up rapid sequencing and assembly of many global SARS-CoV-2 genomes worldwide. Pathogen genomics is an effective tool not only in monitoring the ongoing pandemic but also in effective vaccine development. Genomic data contributed by various laboratories across the world provides a platform to track down the origin and events of community transmission of the virus in their countries [21]. Genomic studies have already proved crucial in contact tracing of infection and would become more important in the second wave of infection after the release of lockdown in the ongoing covid19 pandemic [22]. In this study, we analyzed 121 whole genomes of SARS-CoV-2 from India to infer the phylogeography of SARS-CoV-2 within India, infection genomics to estimate the reproduction number (R0) and infection rate and the past and present evolutionary trajectory of SARS-CoV-2.

## Materials & methods

Whole-genome sequence information on SARS-CoV-2 isolates from India, available in the GISAID database until 4 May 2020, was retrieved. Out of 286 SARS-CoV-2 genomes, samples with missing information and gaps were discarded to arrive at a total of 219 Indian SARS-CoV-2 genomes. These genomes were aligned using Clustal Omega [23] and the number of haplotypes was determined using DnaSP v6 [24]. Based on the emergence of 138 haplotypes, the resulting dataset was further used for downstream analyses. In evolutionary studies, phylogenetic networking is becoming a method of choice for reconstructing evolutionary pathways in many species. The Median-Joining Network (MJN) is one such algorithm developed to reconstruct the unambiguous evolutionary history of species [25]. An MJN was constructed with 138 genomes covering all major states of India (Supplementary Table 1). The birth–death serial model (BDSIR), as implemented in the BEAST2 package, was used to estimate the effective R0 of SARS-CoV-2 in India [26, 27]. In BEAST2, an HKY nucleotide substitution model with a gamma category count of 4, a relaxed lognormal clock with a clock rate of 8.3E-5 subs/site/month corresponding to 1 × 10^-3^ subs/site/year [25] were applied. In Markov Chain Monte Carlo (MCMC) analysis, parameters were sampled every 1000 generations over a total of 10 million generations. The basic R0 and BecomeUinfectiousRate were set with the mean distribution to 18.00, assuming a mean recovery time between 18 and 20 days. BEAST v1.10.4, with similar settings to those of the HKY nucleotide substitution model with a gamma category count of 4, a relaxed lognormal clock, and a clock rate of 8.3E-5 subs/site/month corresponding to 1 × 10^-3^ subs/site/year [28] was utilized to reconstruct the evolutionary dynamics of SARS-CoV-2. The tree prior was set to the coalescent exponential growth to calculate the growth rates of the virus in India. The effective sample sizes and 95% highest posterior density intervals for parameters like the basic R0, BecomeUinfectiousRate, growth rate and the demographic reconstruction of growth rates with exponential growth priors were inspected using Tracer v1.7.0. The trees file was summarized in TreeAnnotator by setting the burnin percentage to ten and the target tree type to maximum clade creditability tree, while the node heights were set to mean heights. The time-scaled Maximum Clade Credibility tree based on MCMC analysis of the 138 SARS-CoV-2 genomes was visualized in the FigTree v1.4.4 tree viewer. The Bayesian Skyline Plot (BSP) method as implemented in BEAST v2.2.0 was used to estimate the effective population size (Ne) for these 138 Indian isolates (Supplementary Table 2). The stamped-date method with HKY nucleotide substitution as a model coupled with 4 gamma category and coalescent Bayesian skyline tree priors were set for the analysis. The MCMC chain length of 10 million steps was applied, the first 10% were discarded as burn-in, and a strict clock rate of 8.33E-5 subs/per/site/per month was used. The log file and tree log file were analyzed to draw the BSP in Tracer v1.7.0.

## Results

**Figure 1.**
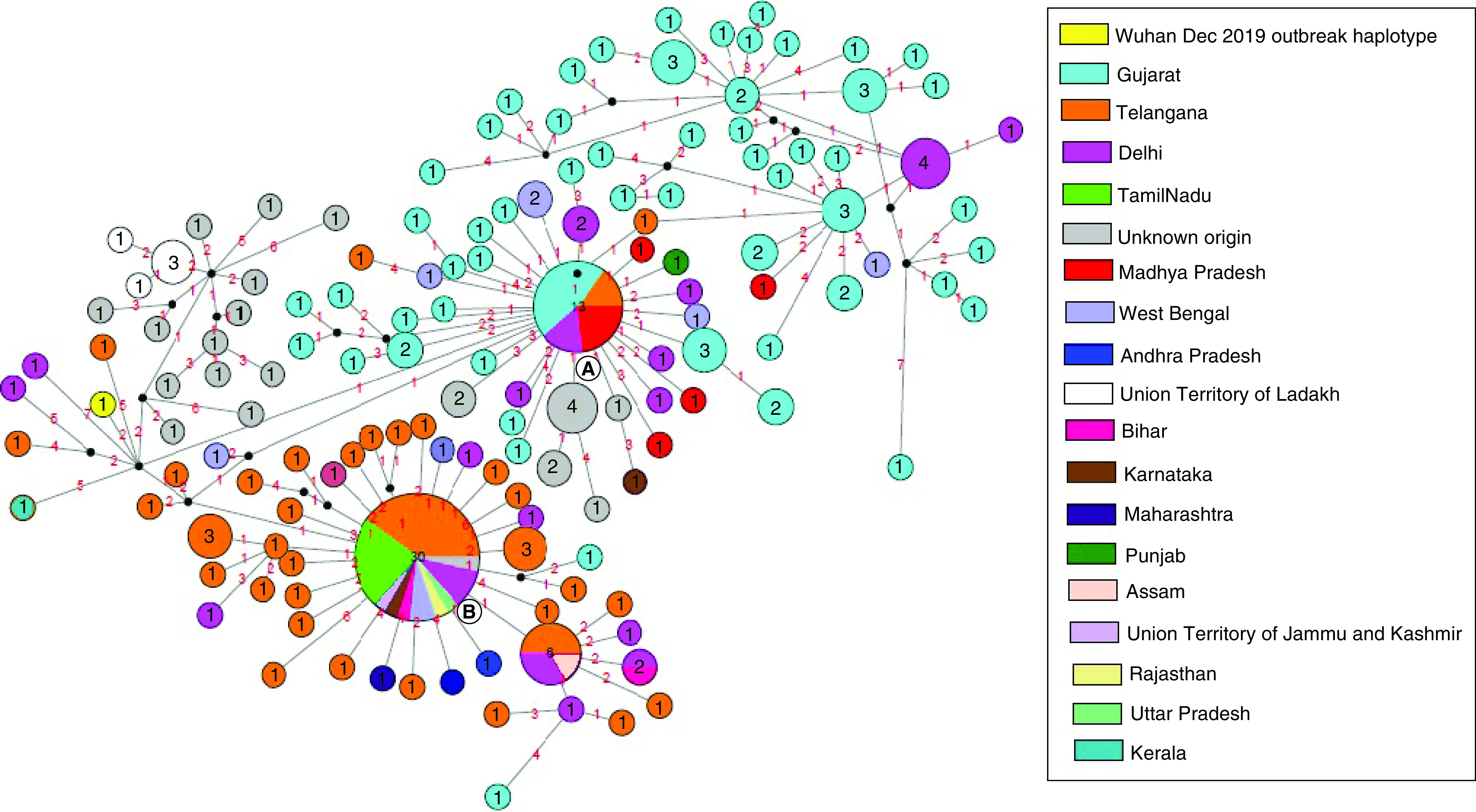
Median-joining network constructed out of 138 Indian haplotypes shows the occurrence of ancestral clusters alongside their newly mutated daughter clusters and haplotypes. The median joining network inferred two main clusters marked as ‘**A**’ and ‘**B**’ showing linkage with the Wuhan, China outbreak haplotype. Circled areas are proportional to the number of shared haplotypes and numbers inside the circle illustrate the number of haplotypes; each number in red on the links represents the position number of the mutated nucleotide. The sequence range under consideration is 56–29,797, with nucleotide position numbering according to the Wuhan, China EPI_ISL_406798 reference sequence. For MJN, Network5011CS (www.fluxusengineering.com/) was used with the parameter epsilon set to zero. MJN: Median-joining network; np: Nucleotide position.

**Figure 2.**
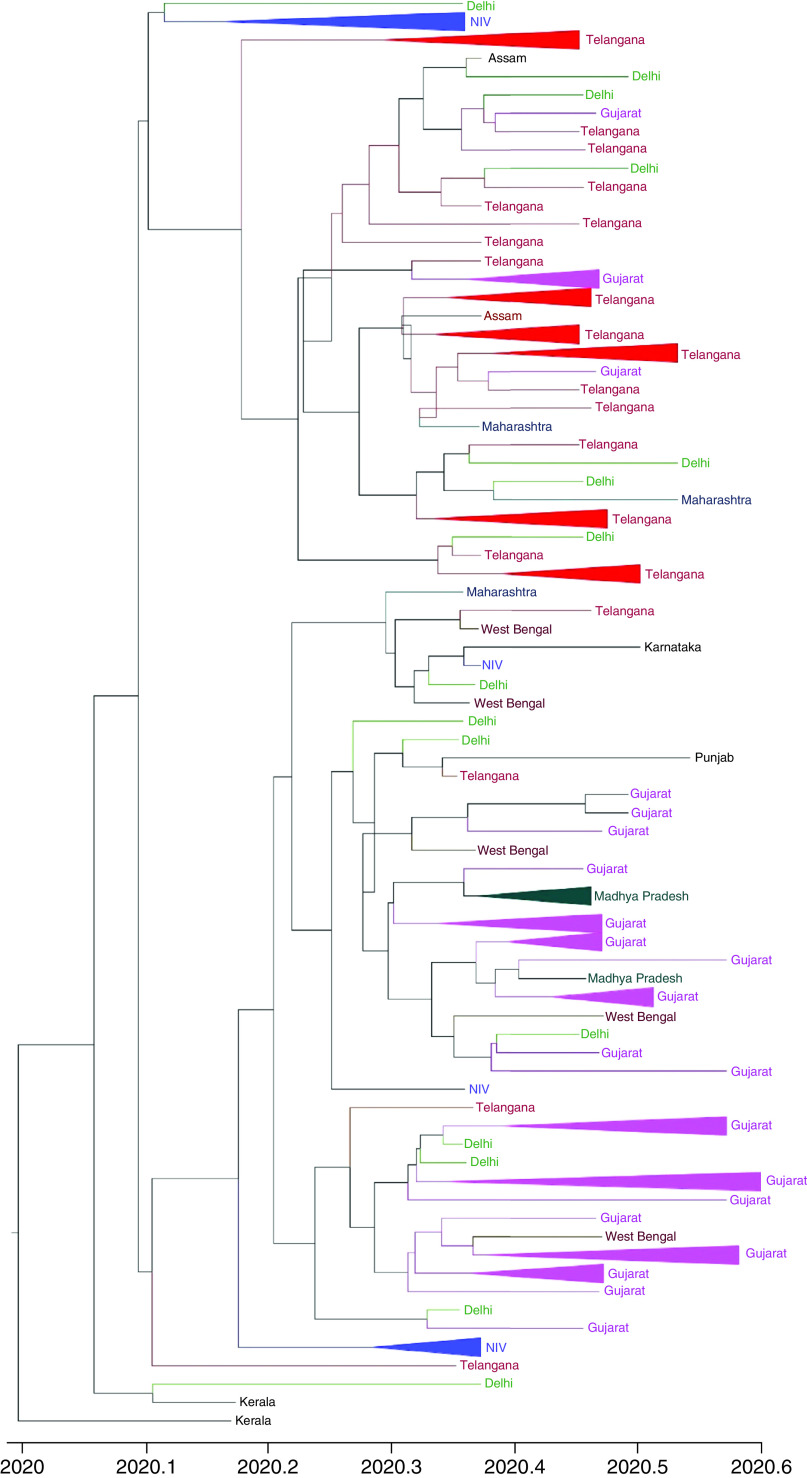
The Maximum clade credibility tree based on Markov Chain Monte Carlo analysis of the 138 Indian SARS-CoV-2 genomes with an exponential growth tree prior.

**Figure 3.**
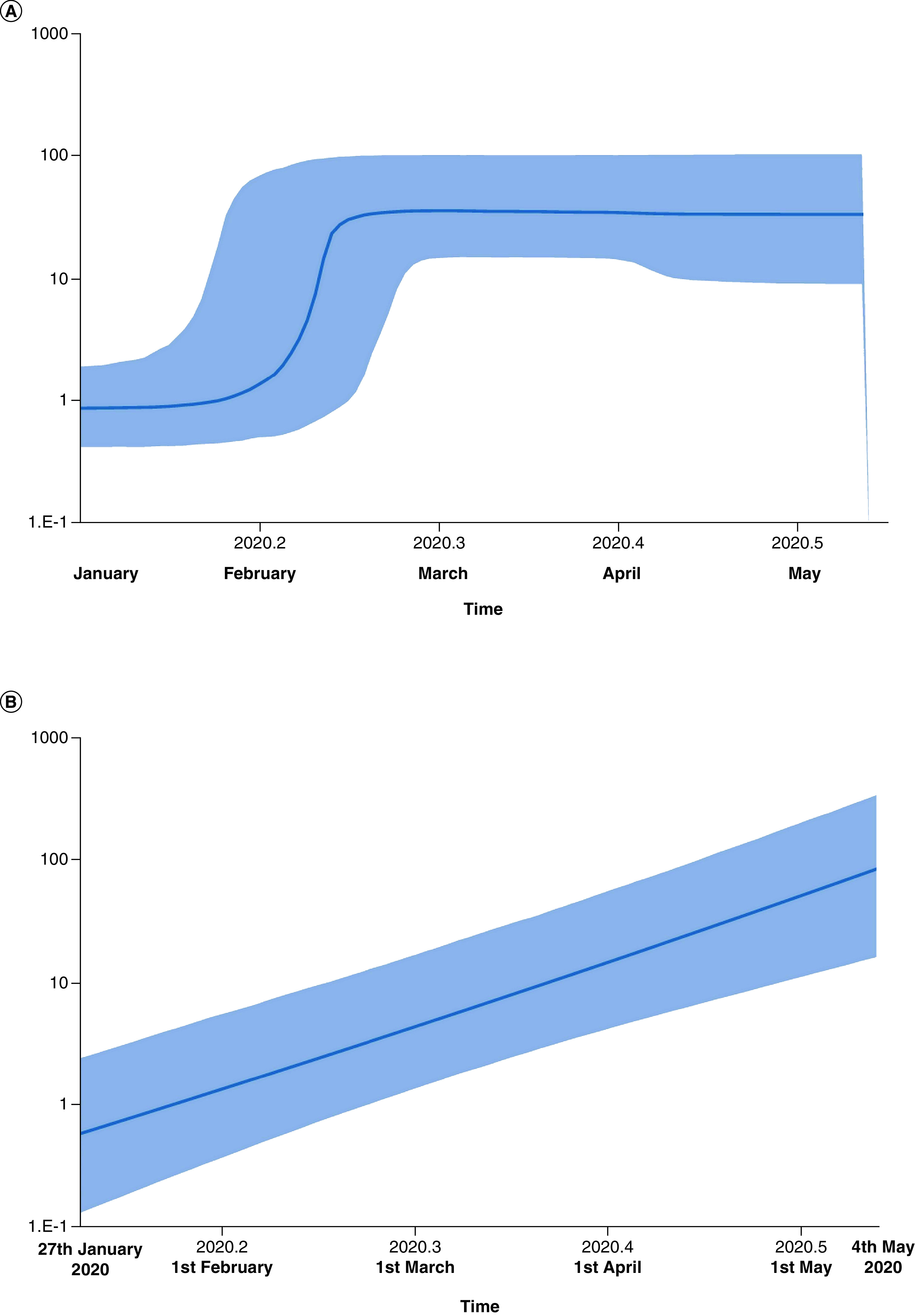
Population dynamics of SARS-CoV-2 in India. Bayesian skyline plots **(A & B)** constructed using 138 Indian isolates by both coalescence Bayesian skyline and exponential growth tree priors depicts the effective population size on the Y-axis, while the X-axis denotes the timeline in months. Ne: Effective population size.

The MJN showed the occurrence of ancestral clusters alongside their newly mutated daughter clusters and haplotypes. The network was defined by two main clusters marked as ‘A’ and ‘B’ (Figure 1). Both A and B clusters showed linkage with the Wuhan, China outbreak haplotype (EPI_ISL_406798) of 26 December 2019. Cluster A, which was dominant in Gujarat, differed by a single median vector from the Wuhan haplotype as compared with cluster B, dominant in south India, which differed by two median vectors. In a biological network, median vectors are interpreted either as unsampled or extinct individuals. Based on this relationship, cluster A was considered as the ancestral node. Genomes of unknown origin contributed by National Institute of Virology, Pune showed greater affinity with SARS-CoV-2 genomes from Wuhan and Ladakh and were linked to ancestral cluster A. However, genome isolates from West Bengal illustrated affinities with both clusters. Of note, haplotypes from the worst hit Maharashtra state, including Mumbai, exhibited closer relatedness to cluster B. Further, the SARS-CoV-2 haplotypes from Delhi were more widespread in distribution, while those from Madhya Pradesh displayed closer relatedness to ancestral cluster A. Many daughter haplotypes accumulated 1–4 mutations that were derived from clusters A and B. In one instance in the network, a daughter cluster derived from cluster B illustrated sharing of haplotypes from Delhi, Telangana and Assam and can potentially be considered as a third minor subcluster. This subcluster further showed the emergence of newer haplotypes by accumulating mutations in the range of 2–3 with the sole representative from Gujarat. Based on this median joining network, autochthonous and community transmission of the virus in India cannot be ruled out. Similarly, the Time-Scaled Maximum Clade Credibility tree based on MCMC analysis with an Exponential Growth tree prior reveals the basal position of the haplotype from Kerala (Figure 2). The tree shows the splitting of haplotypes into two major clades, i.e., Telangana and Gujarat, whereas the samples contributed by the National Institute of Virology, Pune, Maharashtra displayed monophyletic status with the isolates from Telangana (Figure 2). The effective R0 of SARS-CoV-2 in India was found to be 3.683 (95% highest posterior density [HPD] interval: 2.411, 5.401). The estimated mean infectious rate, which is the actual time needed for recovery after the infection, was estimated as 7.44 (95% HPD interval: 4.51, 9.99), which roughly corresponds to approximately 40–50 days. The current population dynamics of SARS-CoV-2 in India from the last week of January (27 January 2020) to the first week of May (4 May 2020) was plotted using a BSP. The population dynamics of Indian isolates exhibited a sigmoidal type of distribution, with exponential growth of the Ne starting from the last week of January to the second week of February (Figure 3A). The pandemic peaked in India around the second week of February and plateaued between the last week of February and 4 May 2020. However, growth rate curve reconstruction using an exponential growth tree prior depicted a continuous increase in the effective population from the last week of January–4 May 2020 (Figure 3B).

## Discussion

Our phylodynamic analysis confirms the high effective R0 and deaths recorded in India during this period. Most probably, the occurrence of a plateau phase in the BSP after the last week of February resulted from the nationwide lockdown and social distancing measures (Figure 3A). Likewise, some findings also suggest that exposure to high temperatures also contributed to the lowering of activity and the lifespan of SARS-CoV-2 [29–31]. In another recently published study, physical distancing measures taken in Wuhan, China beginning in April played a significant role in lowering the reproductive number of virus [32]. Exponential growth was also confirmed in the population dynamics, which was congruent with the continuous rise of SARS-CoV-2 infections in India.

## Conclusion

To conclude, our study provides baseline genome-based phylodynamic information, highlighting genetic affinities between viral isolates sequenced from the major states of India. In the coming days, sequencing and analyses of greater numbers of SARS-CoV-2 genomes from India would help in dealing with the second wave of community transmission after relaxation of the lockdown. At the same time, genomic information produced through such studies can also be utilized to fill the gaps created due to unrealistic assumptions, lack of contact tracing, sampling errors and limited diagnostic testing.

**Summary points**

- The research presented in this study cast light on the phylogenomics and phylodynamics of SARS-CoV-2 genomes retrieved from India.
- A total of 286 SARS-CoV-2 whole genomes deposited from 26 December 2019 to 4 May 2020, representing all major regions of India, were analyzed.
- Out of 286 retrieved whole genomes, a total of 138 haplotypes were identified and used to build a phylogenetic network using the birth-death serial model (BDSIR) package of BEAST2. The reproduction number (R0) was also calculated using the same dataset.
- The population dynamics were also investigated using the stamp date method of constant coalescence as well as exponential growth models as implemented in BEAST2 and BEAST 1.10.4.
- Our median-joining network analysis confirms the dual ancestry of viral haplotypes in India. The phylodynamic analysis validates the high basic R0 of SARS-CoV-2 and an exponential rise in the effective population size of Indian isolates.
- The high basic R0 and the exponential rise in the effective population size of Indian isolates predicted by the Bayesian model highlight the possibility of community transmission of SARS-CoV-2 in India.
- Following relaxation of the lockdown in India and considering the possibility of a second wave of disease transmission, maintaining strict track of infection using genome analysis would aid in the proper management of SARS-CoV-2.
